# Killer cell immunoglobulin-like receptor (KIR) alleles suggested to be associated with myalgic encephalomyelitis/chronic fatigue syndrome (ME/CFS)

**DOI:** 10.1016/j.bbi.2025.106098

**Published:** 2025-08-31

**Authors:** Donia Jamal Ramadan, Katherine M. Kichula, Sudan Tao, Timothy Porfilio, Asgeir Lande, Øystein Fluge, Olav Mella, Elin Bolle Strand, Ola Didrik Saugstad, Paul J. Norman, Benedicte A. Lie, Marte K. Viken

**Affiliations:** a Department of Medical Genetics, University of Oslo and Oslo University Hospital, Oslo, Norway; b Institute of Clinical Medicine, University of Oslo, Oslo, Norway; c Department of Biomedical Informatics, University of Colorado School of Medicine, Aurora, CO, USA; d Department of Immunology and Microbiology, University of Colorado School of Medicine, Aurora, CO, USA; e Department of Oncology and Medical Physics, Haukeland University Hospital, Bergen, Norway; f Department of Clinical Sciences, University of Bergen, Bergen, Norway; g CFS/ME Senteret, Oslo University Hospital, Aker, Oslo, Norway; h Department of Pediatric Research, University of Oslo and Oslo University Hospital, Oslo, Norway; i Department of Immunology, University of Oslo and Oslo University Hospital, Oslo, Norway

**Keywords:** ME/CFS, KIR, HLA, Association

## Abstract

Myalgic encephalomyelitis/chronic fatigue syndrome (ME/CFS) is a chronic and debilitating disease with unknown cause. Involvement of infection and immune dysregulation has been suggested, including changes in immune cell subsets and abnormal functions of natural killer (NK) cells. The regulatory NK cell receptors, killer cell immunoglobulin-like receptors (KIR) have previously been investigated in small cohorts of ME/CFS patients with conflicting results regarding gene content. Here, we studied *KIR* genes also at the allelic level using high-resolution sequencing, in 418 ME/CFS patients and 473 healthy controls. Human leukocyte antigen (HLA) class I genotype data were included for KIR ligand annotation. Our healthy control data represent *KIR* frequencies for a Norwegian population, which have not previously been reported. We found no association between ME/CFS and *KIR* gene content or copy number variations. However, our data suggested that specific *KIR* alleles at loci encoding inhibitory receptors were associated with ME/CFS, which was further supported by allelic haplotype analyses. Three alleles were more frequent in patients, i.e. KIR3DL3*002 (OR = 1.43, 95 % CI (1.09–1.86), p = 0.009), KIR3DL1*020 (OR = 2.20, 95 % CI (1.19–4.06), p = 0.01) and KIR3DL2*009 (OR = 1.56, 95 % CI (1.09–2.23), p = 0.01), while two alleles had a reduced patient frequency, i.e. KIR3DL3*013 (OR = 0.60, 95 % CI (0.42–0.86), p = 0.005) and KIR3DL2*010 (OR = 0.46, 95 % CI (0.30–0.71), p = 0.0005). Our data support an involvement of NK cells in ME/CFS.

## 1. Introduction

Myalgic encephalomyelitis/chronic fatigue syndrome (ME/CFS) has been reported to affect between 17–24 million people globally, giving a population prevalence of approximately 1 % (Lim et al., 2020), but this could be even higher (Vardaman and Gilmour, 2025). The prevalence in women is 1.5–2-fold higher than men (Lim et al., 2020). The disease is characterized by post-exertional malaise (PEM), cognitive impairment, sleep disturbance, joint- and muscle pain, and flu-like symptoms with varying severity (Arron et al., 2024). The exact cause of ME/CFS is unknown, and there are no diagnostic biomarkers. Several diagnostic criteria exist and have been used through the years (Arron et al., 2024), and the disease is considered to have a heterogeneous phenotype. Many ME/CFS patients report an infection-triggered onset, and sporadic cases of ME/CFS have been reported following outbreaks of infectious diseases (Underhill, 2015; Hanson, 2023). Family studies suggest that ME/CFS has a heritable predisposition, with familial occurrence, higher concordance rates in monozygotic than dizygotic twins and increased relative risk in first degree relatives (Underhill, 2015).

Several studies have indicated natural killer (NK) cell dysfunction in ME/CFS, showing reduced cytotoxicity and skewing of NK cell subsets (Klimas et al., 1990; Hardcastle et al., 2015; Brenu et al., 2014; Brenu et al., 2010; Fletcher et al., 2010; Tirelli et al., 1994). NK cells are important in the early host defense against infectious agents, acting as cytotoxic lymphocytes that mediate lysis of virus infected and tumorigenic cells. NK cell functions are regulated by numerous receptors, including the inhibitory and activating killer cell immunoglobulin-like receptors (KIR), through their interactions with their specific human leukocyte antigen (HLA) class I ligands (Vivier et al., 2011). KIR-HLA interactions are also important for the education of NK cells, giving them the capability to mediate cell lysis of diseased cells but avoid killing healthy cells (Anfossi et al., 2006; Vivier et al., 2011). Several *KIR* genes have shown association with autoimmune diseases, e.g. *KIR3DL2* in susceptibility to spondylarthritis (Chan et al., 2005; Bowness et al., 2011) and *KIR2DS1* and/or *KIR2DS2* in susceptibility to psoriatic arthritis (Nelson et al., 2004). *KIR* genes are highly polymorphic, like the *HLA* loci, which affects the KIR function (Pollock et al., 2022).

Only two studies have investigated *KIR* genes in ME/CFS or CFS (Pasi et al., 2011; Huth et al., 2016). Pasi et al. reported a higher carrier frequency of *KIR3DS1* in CFS patients (N = 46) than controls, and that *KIR3DS1* and *KIR3DL1* more often lacked their *HLA* ligands in patients (Pasi et al., 2011). Huth et al. analyzed *KIR* gene content, haplotypes and alleles in 20 ME/CFS patients and 20 non-fatigued controls (Huth et al., 2016) and reported a lower carrier frequency of the telomeric *KIR A/B* haplotype motif combination in patients. Huth et al. did not replicate the reduced carrier frequency of *KIR3DS1* reported by Pasi et al. (Pasi et al., 2011; Huth et al., 2016). Notably, several *HLA* variants have been suggested to be associated with ME/CFS (Smith, 2005; Carlo-Stella et al., 2009; Underhill et al., 2001; Lande et al., 2020; Hajdarevic et al., 2021), including our previously reported association with HLA-C*07:04, which is a C1 ligand for KIR (Lande et al., 2020; Hajdarevic et al., 2021). Given these limited studies of *KIR*, the *HLA* associations and NK cell dysfunction in ME/CFS, comprehensive and statistically powerful *KIR* studies are warranted.

Ours is the largest study, to date, of *KIR* genes in ME/CFS patients (N = 418). In this study, we investigated associations to gene content and copy number, alleles and haplotypes as well as the KIR-HLA ligand combinations.

## 2. Materials and methods

### 2.1. Patient and sample collection

We included 418 Norwegian ME/CFS patients and 473 healthy controls in our study. ME/CFS patient DNA samples were obtained as previously described (Lande et al., 2020; Hajdarevic et al., 2021). All patients had a diagnosis of ME/CFS according to the 2003 Canadian Consensus Criteria (Carruthers et al., 2003). Healthy controls were obtained from the Norwegian Bone Marrow Donor Registry at Oslo University Hospital. Disease severity for ME/CFS patients was assessed according to the DePaul Symptom questionnaire (question 79) (De Meirleir et al., 2010; Jason and Sunnquist, 2018). Written consent was given by participants at inclusion and all samples were de-identified prior to genotyping. The project was approved by the Regional Committees for Medical and Health Research Ethics in Norway (REK 2015/1547).

### 2.2. KIR and HLA genotyping by next generation sequencing

All genomic DNA samples were genotyped by next generation sequencing for all *KIR* and *HLA* genes. The method developed by Norman et al. (Norman et al., 2016) was used for *KIR* gene content, copy number and allele genotyping of ME/CFS patient samples and healthy controls. The method uses probe-based enrichment, with sequencing performed using an Illumina instrument (San Diego, USA). The Pushing Immunogenetics to the Next Generation (PING) pipeline was utilized to analyze the data (Marin et al., 2021). *HLA* genotyping data were obtained from previous studies where the ME/CFS patients had been genotyped using kits from GenDx (Utrecht, The Netherlands) and sequenced using an Illumina Miseq as described (Lande et al., 2020; Hajdarevic et al., 2021), and healthy controls using MIA FORA Immucor kit (Norcross, USA) and Illumina MiniSeq (Hajdarevic et al., 2021; Creary et al., 2021).

### 2.3. KIR and HLA interactions

HLA genotypes were used to identify KIR ligands in patients (N = 404) and controls (N = 473). The Bw4 motif is encoded by the codons in position 77–83 of certain *HLA-A* and *HLA-B* alleles that function as a ligand for KIR3DL1 (Osoegawa et al., 2022). The *HLA-C* genotypes were used to identify the C1 and C2 KIR ligand motif, encoded by codons in position 77 and 80 (Osoegawa et al., 2022). Evaluation of disease association due to KIR-HLA interaction was based on the presence of HLA ligands for their corresponding KIR. HLA ligands included in our analyses were Bw4, C1, C2 and A3/11 (carriers of either HLA-A*03 or – A*11 alleles). The number of Bw4 ligands was assessed based on 1) both *HLA-A* and – *B* genotype, and 2) only *HLA-B* genotype. We further grouped the Bw4 ligand into Bw4^80I^ and Bw4^80T^ carriers. We next investigated KIR-HLA pairs by counting carriers of a specific HLA ligand and the respective KIR (Pollock et al., 2022); i.e. Bw4^+^ and KIR3DL1^+^, Bw4^80I^ and KIR3DL1^+^, Bw4^80T^ and KIR3DL1^+^, C1^+^ and KIR2DL2^+^, C1^+^ and KIR2DL3^+^, C1^+^ and KIR2DS2^+^, C2^+^ and KIR2DL1^+^, C2^+^ and KIR2DL2^+^, C2^+^ and KIR2DS1^+^ and A3/11^+^ and KIR3DL2^+^.

### 2.4. KIR haplotypes

**Fig. 1.**
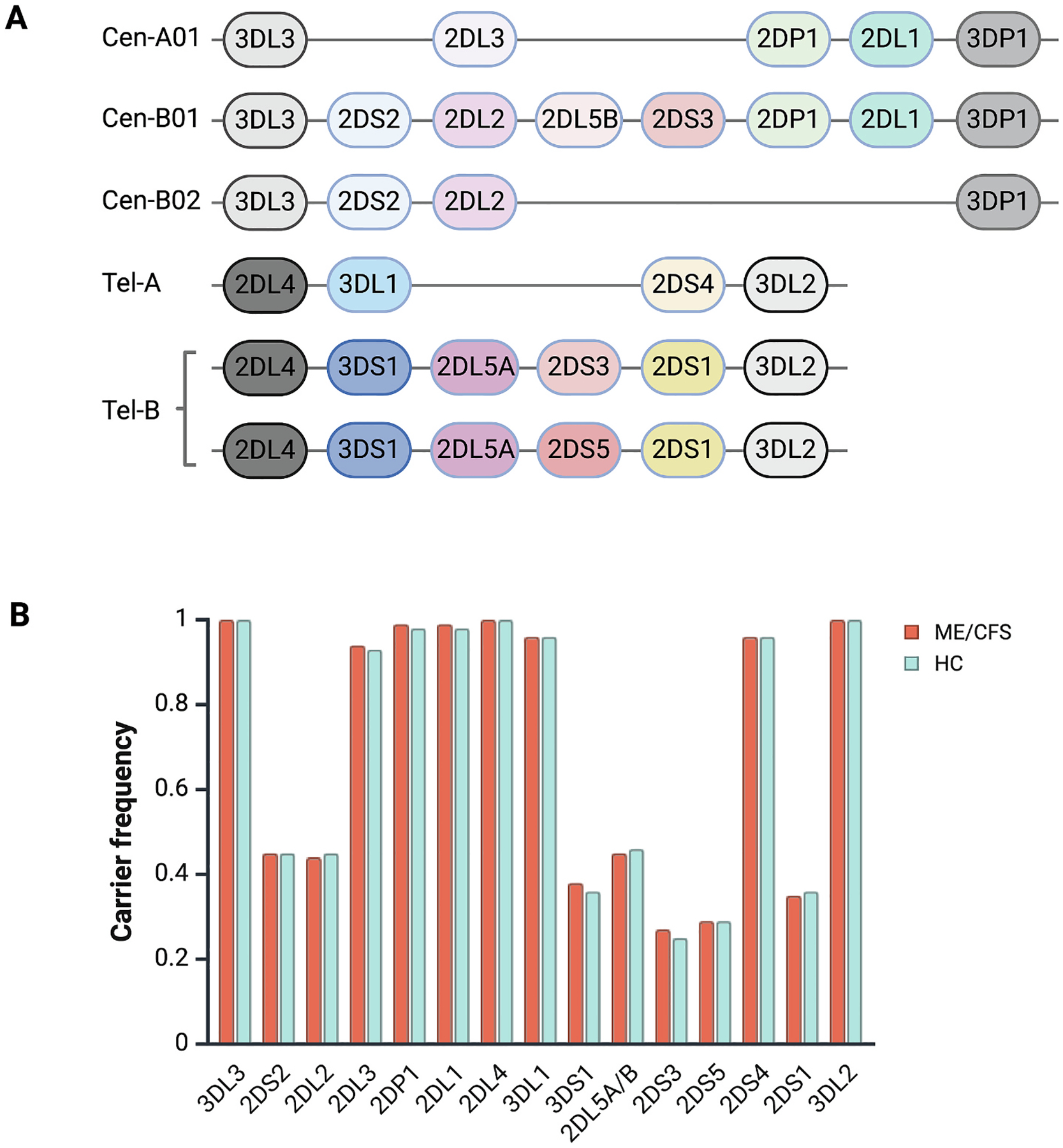
A) A schematic overview of the haplotype motif structure used in this paper (haplotypes with duplicated/deleted regions are not depicted) (Robinson et al., 2012; Pende et al., 2019), and B) Carrier frequencies of KIR genes in ME/CFS patients and healthy controls (HC)). Created with BioRender.com.

*KIR* haplotypes were assigned into haplotype A and haplotype B based on the gene content in each individual (Robinson et al., 2012). More specifically, the following centromeric and telomeric haplotype motifs were assigned (Fig. 1A): cA01, cB01 and cB02 for the centromeric region and tA01 and tB01 for the telomeric region (Pende et al., 2019). We included the genes *KIR3DL3, KIR2DS2*, *KIR2DL2/3*, *KIR2DL5B*, centromeric *KIR2DS3*, *KIR2DP1* and *KIR2DL1* for centromeric haplotypes, and *KIR2DL4*, *KIR3DL1*, *KIR3DS1*, *KIR2DL5A*, *KIR2DS3*, *KIR2DS5*, *KIR2DS4*, *KIR2DS1* and *KIR3DL2* for telomeric haplotypes. Rarer haplotypes with duplicated or deleted genes were categorized into one group. Pinpointing the location of deleted or duplicated genes on the centromeric and telomeric regions can be ambiguous, however, to ensure consistency we rigorously applied the same assignment strategy both for cases and controls. Notably, 10 % of the controls were quality controlled against previous PCR/gel electrophoresis gene content data (Lorentzen et al., 2009) for the haplotype motifs. There was a 96 % concordance, with samples identified with deleted and/or duplicated genes being most difficult to identify using the previous PCR-based data.

### 2.5. Data and statistical analyses

For haplotypes with duplicated alleles, the duplicated alleles were included as distinct loci. Hence, all allele frequency calculations were divided by 2 N for all loci. The *KIR2DL2/3* gene has alleles annotated as *KIR2DL2* and *KIR2DL3*, similarly for *KIR2DS3/5* with *KIR2DS3* and *KIR2DS5*. Allele frequencies were analyzed using Unphased v.3.0.13 (Dudbridge, 2003). Gene absence was included as a distinct allele in all analyses. An expectation–maximization (EM) algorithm was used to estimate maximum-likelihood haplotype frequencies with the Arlequin v3.5 software (Excoffier and Lischer, 2010). Haplotype analyses were performed for all samples separated into a telomeric and a centromeric motif. With 76 *KIR* alleles tested, a Bonferroni corrected significance threshold would be 0.0007. For association analyses, odds ratios (ORs) with 95 % confidence intervals (95 % CI) were calculated with Woolf’s formula comprising Haldane’s correction. Our study has an *a priori* power of > 80 % to detect OR = 1.6, given a risk allele frequency of 0.1, disease prevalence of 1 % and 5 % type I error rate (Purcell et al., 2003).

## 3. Results

### 3.1. Patient and control demographics

An overview of demographics and clinical characteristics of the ME/CFS patients and controls is presented in Table 1. The mean age at inclusion for ME/CFS patients was 39.5 years, with 72.5 % being female and the majority (85.2 %) had a disease duration of more than 5 years. Only 13.1 % of the patients reported comorbidity with autoimmune diseases, with Hashimoto’s thyreoiditis/hypothyreosis being the most commonly reported. Most patients had a high symptom burden (83.7 %), ranging from being bedridden to being able to do light housework, as well as being unable to work the last six months prior to inclusion.

### 3.2. KIR alleles showed signs of association with ME/CFS

Global allelic tests for the *KIR* genes showed significant skewed allelic distributions for the framework genes *KIR3DL3* (p = 0.01) and *KIR3DL2* (p = 0.001). Three alleles were more frequent in ME/CFS (Table 2), i.e. KIR3DL3*002 (OR = 1.43, p = 0.009), KIR3DL1*020 (OR = 2.20, p = 0.01) and KIR3DL2*009 (OR = 1.56, p = 0.01). Two alleles had a reduced frequency among patients; i.e. KIR3DL3*013 (OR = 0.60, p = 0.005) and KIR3DL2*010 (OR = 0.46, p = 0.0005). Notably, the positively associated alleles are found on a haplotype: KIR2DL4*001 ~ KIR3DL1*020 ~ KIR2DS4*001 ~ KIR3DL2*009, also being more prevalent in patients than controls (OR = 2.35, p = 0.008). Supplementary Table 1 shows all haplotypes above 1 % in both cases and controls. None of the remaining alleles showed any significant differences in frequency distribution between cases and controls (Supplementary Table 2).

### 3.3. HLA ligands and KIR-HLA interaction in ME/CFS compared to healthy controls

Next, we wanted to investigate the distribution of *HLA* variants defined as KIR ligands in cases versus controls. There were no significant associations with the number of KIR ligands (Supplementary Table 3). Notably, we did observe a significantly higher presence of Bw4^80I^ carriers in ME/CFS patients compared to controls (OR = 1.54, p = 0.02, Table 2). Furthermore, we analyzed KIR-HLA interactions (Supplementary table 4) and found that carriers of Bw4^80I^ and *KIR3DL1* were more frequent among patients than controls (OR = 1.60, p = 0.01, Table 2).

### 3.4. No significant differences in KIR gene content or haplotype motifs were observed

We found no significant differences in gene content, when defined as presence or absence, between ME/CFS patients and controls (Fig. 1B). Investigating copy numbers, *KIR2DL4* showed the highest variation, however, no significant differences were observed for copy number variation of any *KIR* genes (Supplementary figure 1). The carrier frequencies of genes encoding activating and inhibitory KIR were similar between patients and controls (Supplementary figure 2).

We next defined the haplotype groups (Fig. 1A). There were no significant differences between cases and controls across any of the haplotype A and B motif groups, neither for the centromeric nor telomeric motifs. Carrier frequencies for the haplotype combinations A/A, A/B and B/B, were similar in cases and controls, both for centromeric and telomeric motifs (Supplementary Table 5).

### 3.5. KIR frequencies in the Norwegian population

**Fig. 2.**
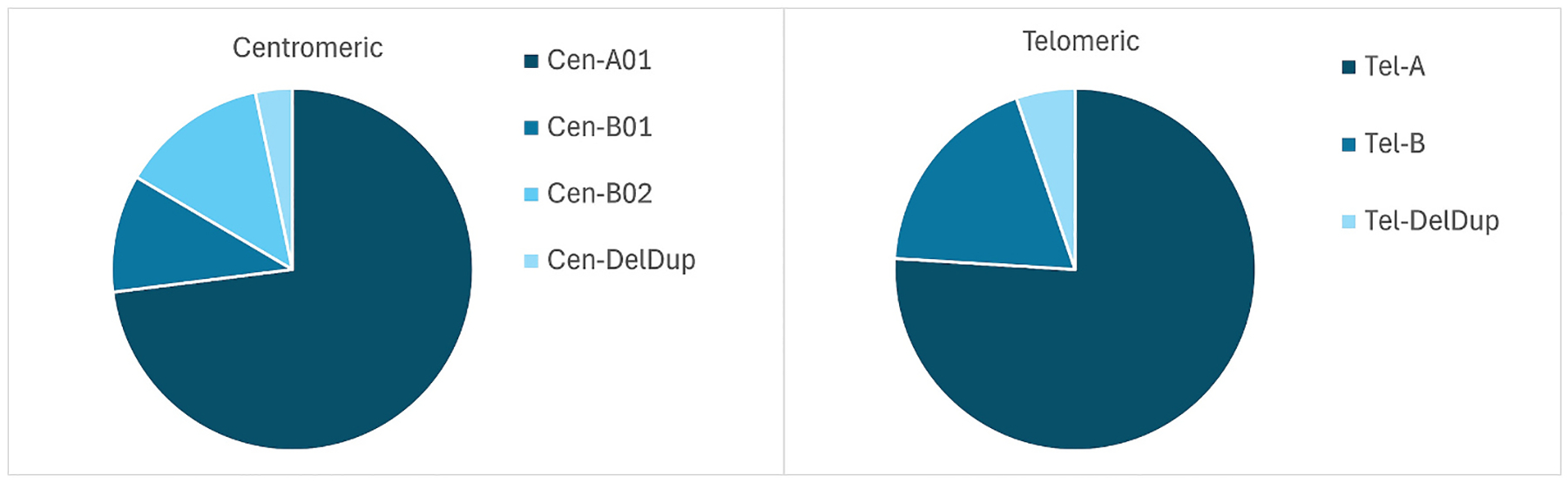
The distribution of haplotype motifs in healthy controls, representative of the Norwegian population.

Our data for the healthy controls also represent *KIR* frequencies in the Norwegian population, which has not previously been published. As expected, the *KIR-A* haplotype motifs, both centromeric and telomeric, were the most common in the Norwegian population (Fig. 2). The allele frequency distributions above 1 % are shown in supplementary Table 2. The *KIR3DL3* locus had the highest number of alleles. Allele-based haplotypes (Supplementary Table 1) showed that the centromeric haplotype KIR3DL3*001 ~ KIR2DL3*002~~KIR2DP1*003 ~ KIR2DL1*002 had the highest frequency with 12.8 %. For the telomeric region, the haplotype KIR2DL4*005 ~ KIR3DS1*013 ~ KIR2DL5A*001 ~ KIR2DS5*002 ~ KIR2DS1*002 ~ 3DL2*007 was the most frequent in healthy Norwegian controls (12. 9 %).

## 4. Discussion

Our results demonstrate that certain alleles of genes encoding inhibitory KIR (*KIR3DL3*, *KIR3DL2* and *KIR3DL1)* could be involved in the development of ME/CFS.

Our associations are reported with uncorrected P values. If considering the study specific Bonferroni corrected significance threshold of 0.0007, only KIR3DL2*010 remains significant (p= 0.0005). Replication in larger cohorts is necessary to validate our findings, as accumulating datasets are essential for establishing robust genetic associations that achieve genome-wide significance. Still, our study represents the largest *KIR* genetic study in ME/CFS (N = 418) performed to date, as previous studies had less than 50 patients (Pasi et al., 2011; Huth et al., 2016). Noteworthy, our ORs are in line with the effect sizes seen in other association studies on multi-factorial and immune-mediated diseases (Wellcome Trust Case Control Consortium, 2007). Our lack of associations with gene content and copy number variation are in agreement with the study by Huth et al. (Huth et al., 2016). However, the higher carrier frequency of the *KIR3DS1* gene among cases reported by Pasi et al. (Pasi et al., 2011), was not replicated by either Huth et al. or us. Huth et al. reported a significantly lower frequency of the telomeric A/B motif in ME/CFS compared to non-fatigued controls (Huth et al., 2016), which was not observed in our larger study.

All three *KIR* loci with associated alleles in our study encode inhibitory receptors, characterized by a long cytoplasmic fragment with an immunoreceptor tyrosine-based inhibitory motif. Both *KIR3DL3* and *KIR3DL2* are framework genes and present in every individual (Parham and Guethlein, 2018), while *KIR3DL1* is carried by 96 % of our population. Concerning the putative role of KIR in ME/CFS, KIR3DL1 expression on NK cells has been reported to be significantly increased in 11 CFS patients with moderate disease severity (Brenu et al., 2013). A later study, however, reported no difference in expression levels of several KIRs, including KIR3DL1, between ME/CFS patients and controls (Brenu et al., 2014). Despite these conflicting findings, it is worth noting that the *KIR3DL1* locus is known to have allelic differential expression (Gardiner et al., 2001). Interestingly, the ME/CFS associated KIR3DL1*020 is known as a highly expressed *KIR* allele (Yawata et al., 2006; Thomas et al., 2008). In contrast, another of our ME/CFS associated alleles, KIR3DL2*009, has been reported to have lower expression compared to other KIR3DL2 allotypes (Yawata et al., 2006). These two alleles, KIR3DL2*009 and KIR3DL1*020, appeared together on a telomeric KIR-A haplotype, in line with the reported linkage disequilibrium (LD) of D́=0.85 in a North American population (Amorim et al., 2021). Hence, the association with KIR3DL1*020 is likely due to linkage disequilibrium with KIR3DL2*009, but these could not be distinguished in our dataset (data not shown). A larger genetic association study designed to fine-map the region would be necessary to resolve this.

The Bw4 allotype is a ligand for KIR3DL1, one of the genes we found to be associated with ME/CFS. In addition to the allelic association, we observe a higher carrier frequency of both Bw4^80I^ and *KIR3DL1*. This specific KIR-HLA interaction has previously been implicated in psoriasis and autoimmune hepatitis (Ahn et al., 2016; Umemura et al., 2019). However, in psoriasis the reported association was with low expressed *KIR3DL1* alleles such as KIR3DL1*005 (Ahn et al., 2016); which is in contrast to our associated high expression allele, KIR3DL1*020. Although the same genes are involved in different diseases, the biological mechanisms and pathways may differ. Interestingly, several KIRs are also expressed on subsets of T cells, even though they were first identified from NK cells (Young and Uhrberg, 2002). For example, KIR3DL3, which we found to be associated with ME/CFS, has recently been shown to be enriched in γδ and CD8 + T cells and not NK cells (Palmer et al., 2023). Furthermore, in contrast to KIR3DL1 and KIR3DL2 which have HLA class I variants as ligands (Pollock et al., 2022), KIR3DL3 has HHLA2 (Human endogenous retrovirus-H Long repeat-associating 2) as ligand (Palmer et al., 2023). KIR3DL2 also have other ligands than HLA-A3 and – A11, these include HLA-B27-free heavy chains and microbial CpG-oligodeoxynucleotide (ODN) (Pugh et al., 2021; Sivori et al., 2010). Binding of CpG-ODN to KIR3DL2 will induce down-modulation of KIR3DL2 on the cell surface and internalization and translocation of CpG-ODN to endosomes containing toll-like receptor 9 which promote NK cell cytotoxicity by cytokine release (Sivori et al., 2010). Hence, KIR3DL2 can influence the induction of rapid response to microbes by acting as transporters of CpG-ODNs. As many ME/CFS patients report an infection-triggered onset, it is interesting that common viruses like the human cytomegalovirus (HCMV) can shape the NK cell receptor repertoire, including KIRs, and lead to adaptive-like expansion of NK cells giving them distinct functional capabilities (Béziat et al., 2013; Schlums et al., 2015). Another virus commonly infecting humans are Epstein-Barr virus (EBV), where NK cells also seem to have an important role, and where a recent study have shown that differentiated KIR-positive NK cells are those that respond better against cells with lytic EBV replication (Desimio et al., 2024). Both KIR and HLA are involved in infection control, e.g. for Covid-19, where it is suggested that the activating receptor encoded by *KIR2DS2* with its cognate HLA-C1 ligand may have a protective effect against severe infection (Littera et al., 2021). Taken together, this could imply that KIRs have multiple and/or variable roles in different diseases’ development and progression. Furthermore, our KIR and previous HLA associations with ME/CFS may support that common infections could trigger the disease.

Finally, our study also provides *KIR* frequencies in healthy controls from the Norwegian population, which thus can be used as references for future *KIR* studies of other diseases. The Norwegian frequencies of the most common alleles for each *KIR* gene were similar to those previously reported in populations of European origin (Tao et al., 2021; Wagner et al., 2018).

In summary, our data suggest associations between ME/CFS and certain alleles at genes encoding inhibitory KIRs, which warrants replication in larger cohorts.

## Supplementary Material

1

2

## Figures and Tables

**Table 1:** Demographic and clinical characteristics of ME/CFS patients and healthy controls.

	Patients	Controls
Mean age, years (min, max)^a,b^	39.5 (17,79)	37.6 (23,55)
Percentage females^a,b^	72.5	63.2
Disease onset triggered by infection (self-reported)^c^	72.9	–
Comorbidity with autoimmune diseases (self-reported)^d^	13.1	–
**Disease duration, percentage of patients** ^e^		
1–5 years	14.8	–
5–10 years	44.4	–
10–15 years	27.4	–
>15 years	13.4	–
**Disease severity, measured by activity assessment in DSQ79** * **, percentage of patients** ^f^		
Cat 1: Bedridden	12.7	–
Cat. 2: Strictly housebound	29.6	–
Cat. 3: Light housework	41.4	–
Cat. 4: Able to work part time	11.6	–
Cat. 5: Able to work full time	0.8	–
Cat. 6: Handling some family obligations	0.3	–
Cat. 7: Handling work and family obligations	0.0	–

* **DePaul Symptom Questionnaire, question no. 79** (De Meirleir et al., 2010; Jason and Sunnquist, 2018)

Cat.1: I am not able to work or do anything, and I am bedridden.

Cat.2: I can walk around the house, but I cannot do light housework.

Cat.3: I can do light housework, but I cannot work part-time.

Cat.4: I can only work part time at work or on some family responsibilities.

Cat.5: I can work full time, but I have no energy left for anything else.

Cat.6: I can work full time and finish some family responsibilities, but I have no energy left for anything else.

Cat.7: I can do all work or family responsibilities without any problems with my energy.

a Valid number of patients: 418

b Valid number of controls: 473

c Valid number of patients: 402

d Valid number of patients: 398

e Valid number of patients: 277

f Valid number of patients: 355

**Table 2:** Significant allelic associations between ME/CFS and KIR loci or ligands.

Global allelic associations	Cases (N = 418)	Controls (N = 473)		*P*
*KIR3DL3*	–	–	–	0.01
*KIR3DL2*	–	–	–	0.001
Allelic associations	Frequency in cases (2N = 836)	Frequency in controls (2N = 946)	OR (95 % CI)	*P*
KIR3DL3*002	0.17 (139)	0.12 (116)	1.43 (1.09–1.86)	0.009
KIR3DL3*013	0.06 (48)	0.09 (88)	0.60 (0.42–0.86)	0.005
KIR3DL2*009	0.09 (75)	0.06 (56)	1.56 (1.09–2.23)	0.01
KIR3DL2*010	0.04 (30)	0.08 (71)	0.46 (0.30–0.71)	0.0005
KIR3DL1*020	0.03 (29)	0.02 (15)	2.20 (1.19–4.06)	0.01
Allelic haplotype associations*	Estimated frequency in cases (2N = 836)	Estimated frequency in controls (2N = 946)	OR (95 % CI)	*P*
KIR2DL4*011 ~ KIR3DL1*005 ~ KIR2DS4*010 ~ KIR3DL2*001	0.151	0.117	1.34 (1.02–1.76)	0.04
KIR2DL4*001 ~ KIR3DL1*020 ~ KIR2DS4*001 ~ KIR3DL2*009	0.035	0.015	2.35 (1.26–4.40)	0.008
KIR2DL4*011 ~ KIR3DL1*005 ~ KIR2DS4*010 ~ KIR3DL2*010	0.01	0.044	0.46 (0.26–0.80)	0.006
KIR3DL3*002 ~ KIR2DL3*001 ~ KIR2DP1*002 ~ KIR2DL1*003	0.133	0.09	1.55 (1.15–2.08)	0.004
KIR3DL3*014 ~ KIR2DS2*001 ~ KIR2DL2*003	0.019	0.035	0.54 (0.30–0.97)	0.04
KIR3DL3*013 ~ KIR2DL3*001 ~ KIR2DP1*002 ~ KIR2DL1*003	0.014	0.03	0.46 (0.24–0.91)	0.02
HLA KIR ligand carriers^#^	Carrier frequency in cases (N = 404)	Carrier frequency in controls (N = 473)	OR (95 % CI)	*P*
Bw4^80I^	0.205 (83)	0.144 (68)	1.54 (1.08–2.18)	0.02
KIR-HLA interactions^#^	Frequency in cases (N = 404)	Frequency in controls (N = 473)	OR (95 % CI)	*P*
KIR3DL1 and Bw4^80I^	0.20 (81)	0.135 (64)	1.60 (1.12–2.28)	0.01

* Significant alleles at the allelic level are marked bold.

# N = Bw4^80I^ annotations were HLA-B based.

## Data Availability

The authors do not have permission to share data.

## Appendix A. Supplementary data

Supplementary data to this article can be found online at https://doi.org/10.1016/j.bbi.2025.106098.
