# Supplementary material for: Killer cell immunoglobulin-like receptor (KIR) alleles suggested to be associated with myalgic encephalomyelitis/chronic fatigue syndrome (ME/CFS)
Source: Brain Behav Immun. Author manuscript; Available in PMC 2026 May 26. (PMC13201895; doi:10.1016/j.bbi.2025.106098)
Supplement: 1 [file NIHMS2175711-supplement-1.pdf]

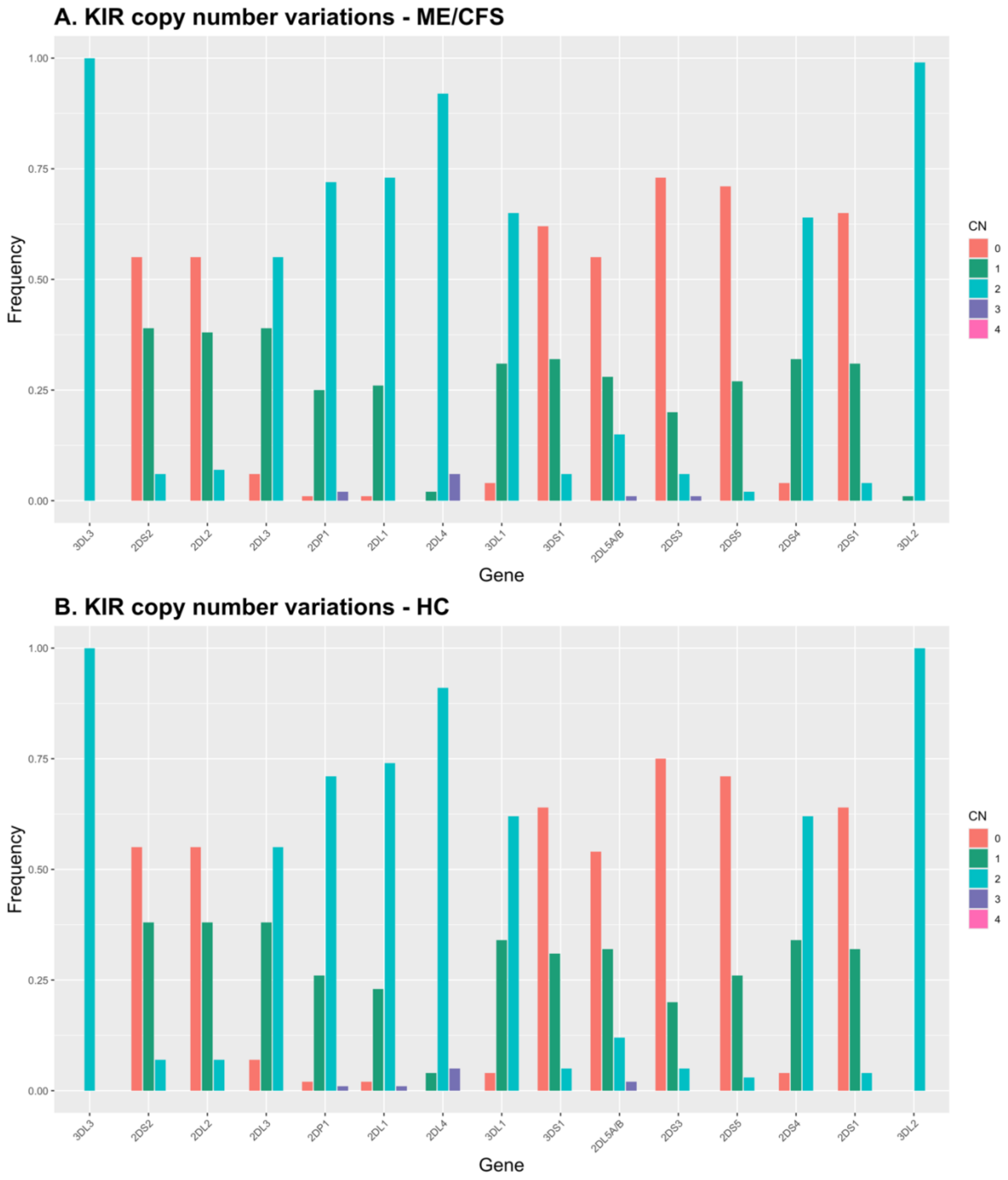

Supplementary figure 1: *KIR* copy number variation in ME/CFS and healthy controls. Frequencies for carriers of varying copies (0-4) of each *KIR* are displayed for cases and controls.

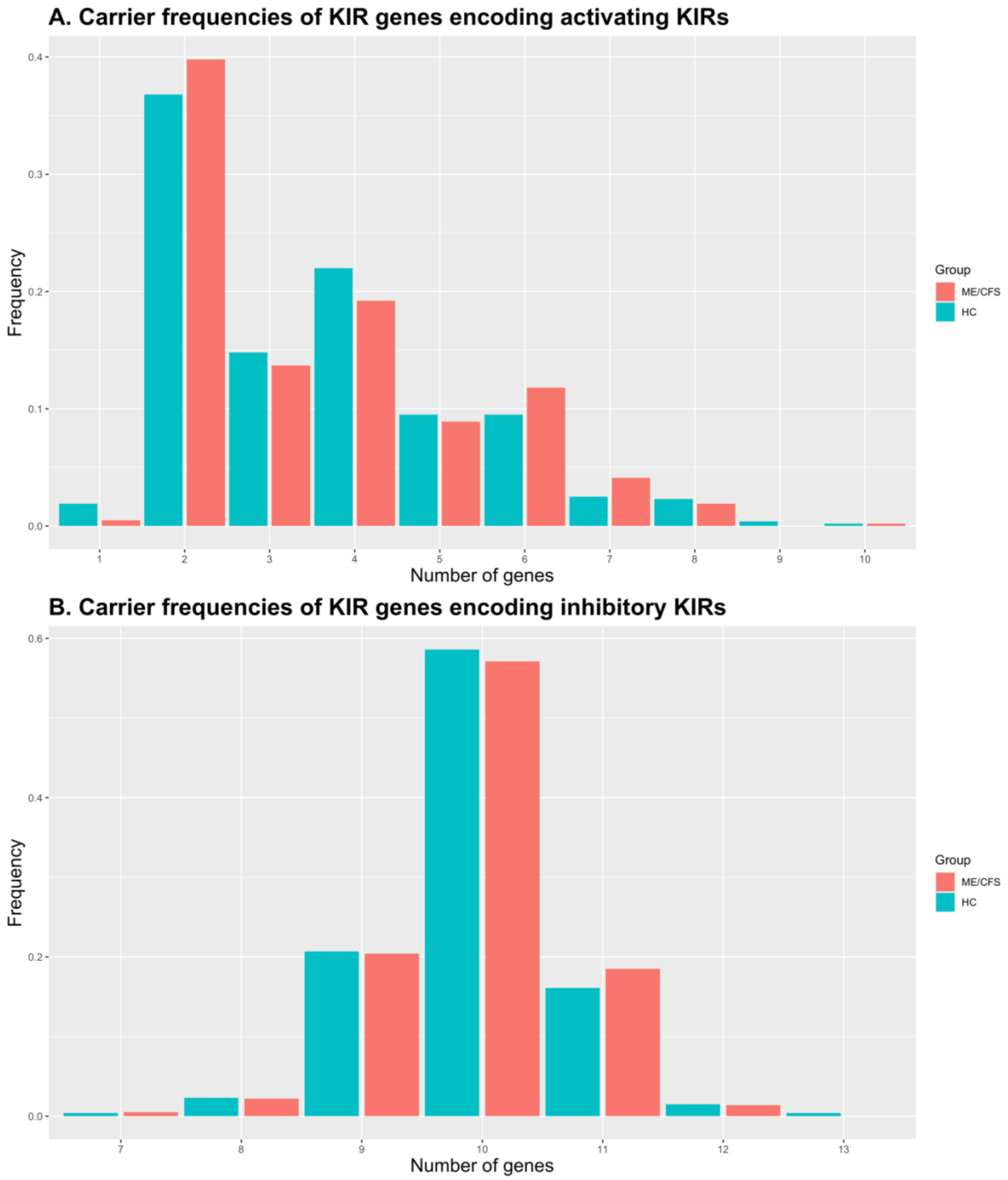

Supplementary figure 2: Carrier frequencies of *KIR* genes encoding activating and inhibitory receptors. Frequencies for carriers of varying number of *KIR* genes are displayed for cases and controls.
