# Supplementary material for: Killer cell immunoglobulin-like receptor (KIR) alleles suggested to be associated with myalgic encephalomyelitis/chronic fatigue syndrome (ME/CFS)
Source: Brain Behav Immun. Author manuscript; Available in PMC 2026 May 26. (PMC13201895; doi:10.1016/j.bbi.2025.106098)
Supplement: 2 [file NIHMS2175711-supplement-2.pdf]

Supplementary table 1: Allele based haplotype frequencies above 1% in both ME/CFS and healthy controls

Centromeric

	KIR gene							Frequency			
**Haplotype**	***3DL3***	***2DS2***	***2DL2/3***	***2DL5B***	***2DS3***	***2DP1***	***2DL1***	**Cases (2N=836)**	**Controls (2N=946)**	**OR (95%CI)**	***P***
**cA01**	*002		3*001			*002	*003	0.133	0.09	1.55 (1.15-2.08)	0.004
**cA01**	*001		3*002			*003	*002	0.115	0.128	0.88 (0.67-1.18)	0.4
**cA01**	*009		3*001			*002	*003	0.099	0.119	0.82 (0.61-1.10)	0.18
**cA01**	*001		3*001			*002	*003	0.068	0.058	1.20 (0.82-1.75)	0.35
**cB02**	*003	*001	2*003					0.041	0.029	1.43 (0.86-2.36)	0.17
**cA01**	*006		3*002			*003	*002	0.04	0.029	1.42 (0.85-2.35)	0.18
**cA01**	*013		3*002			*003	*002	0.038	0.055	0.68 (0.44-1.07)	0.09
**cA01**	*017		3*002			*003	*002	0.036	0.025	1.42 (0.83-2.42)	0.2
**cB01**	*003	*001	2*001	*002	*001	*001	*004	0.035	0.024	1.44 (0.84-2.49)	0.18
**cB01**	*014	*001	2*001	*002	*001	*001	*004	0.027	0.019	1.45 (0.78-2.67)	0.24
**cA01**	*015		3*001			*002	*003	0.022	0.012	1.85 (0.90-3.81)	0.09
**cB02**	*014	*001	2*003					0.019	0.035	0.54 (0.30-0.97)	0.04
**cA01**	*010		3*001			*002	*003	0.017	0.018	0.97 (0.49-1.92)	0.93
**cB02**	*014	*001	2*001					0.017	0.021	0.83 (0.42-1.61)	0.58
**cA01**	*008		3*001			*002	*003	0.014	0.013	1.13 (0.52-2.46)	0.75
**cA01**	*013		3*001			*002	*003	0.014	0.03	0.46 (0.24-0.91)	0.02
**cA01**	*002		3*005			*016	*001	0.013	0.015	0.91 (0.42-1.95)	0.81
**cB01**	*003	*002	2*001	*002	*001	*001	*004	0.013	0.014	0.94 (0.43-2.03)	0.87
**cB02**	*007	*001	2*003					0.012	0.017	0.71 (0.33-1.53)	0.39

Telomeric

	KIR gene								Frequency			
**Haplotype**	***2DL4***	***3DL1***	***3DS1***	***2DL5A***	***2DS3/5***	***2DS4***	***2DS1***	***3DL2***	**Cases (2N=836)**	**Controls (2N=946)**	**OR (95%CI)**	***P***
**tA01**	*011	*005				*010		*001	0.151	0.117	1.34 (1.02-1.76)	0.04
**tB01**	*005		*013	*001	5*002		*002	*007	0.126	0.129	0.98 (0.74-1.29)	0.88
**tA01**	*008	*001				*003		*001	0.114	0.112	1.02 (0.76-1.36)	0.9
**tA01**	*001	*002				*001		*002	0.099	0.11	0.89 (0.66-1.21)	0.46
**tA01**	*001	*015				*001		*002	0.067	0.068	0.99 (0.69-1.43)	0.96
**tA01**	*008	*004				*006		*003	0.055	0.058	0.94 (0.63-1.40)	0.76
**tA01**	*008	*004				*006		*005	0.049	0.049	1.01 (0.66-1.55)	0.96
**tA01**	*001	*008				*003		*009	0.039	0.036	1.10 (0.68-1.78)	0.69
**tA01**	*001	*020				*001		*009	0.035	0.015	2.35 (1.26-4.40)	0.008
**tA01**	*006	*007				*004		*008	0.026	0.039	0.67 (0.40-1.13)	0.14
**tB01**	*005		*013	*005	3*002		*002	*007	0.025	0.024	1.04 (0.58-1.86)	0.91
**tA01**	*008	*001				*003		*011	0.02	0.011	1.90 (0.89-4.06)	0.09
**tA01**	*011	*005				*010		*010	0.01	0.044	0.46 (0.26-0.80)	0.006

Supplementary table 2: *KIR* allele frequencies above 1% in both Norwegian ME/CFS and healthy controls

KIR gene	Frequency			
3DL3	**Cases (2N=836)**	**Controls (2N=946)**	**OR (95%CI)**	***P***
*001	0.22 (186)	0.22 (211)	1.00 (0.80-1.25)	0.98
*002	0.17 (139)	0.12 (116)	1.43 (1.09-1.86)	0.009
*003	0.12 (97)	0.10 (97)	1.15 (0.85-1.55)	0.36
*004	0.02 (13)	0.02 (16)	0.92 (0.45-1.89)	0.83
*006	0.05 (39)	0.04 (37)	1.20 (0.76-1.89)	0.43
*007	0.02 (17)	0.03 (26)	0.74 (0.41-1.36)	0.33
*008	0.02 (13)	0.01 (12)	1.23 (0.57-2.62)	0.60
*009	0.11 (94)	0.14 (132)	0.78 (0.59-1.04)	0.09
*011	0.02 (15)	0.01 (10)	1.68 (0.78-3.65)	0.19
*013	0.06 (48)	0.09 (88)	0.60 (0.42-0.86)	0.005
*014	0.07 (57)	0.09 (83)	0.76 (0.54-1.08)	0.13
*015	0.03 (28)	0.03 (26)	1.22 (0.72-2.09)	0.46
*017	0.04 (33)	0.03 (25)	1.51 (0.90-2.53)	0.12
*020	0.01 (9)	0.02 (16)	0.65 (0.29-1.42)	0.28
2DS2				
*001	0.24 (199)	0.23 (222)	1.02 (0.82-1.27)	0.87
*002	0.02 (16)	0.02 (23)	0.79 (0.42-1.48)	0.46
Neg	0.74 (621)	0.74 (699)	1.02 (0.83-1.26)	0.85
2DL2/3				
*L2:001	0.16 (130)	0.14 (130)	1.16 (0.89-1.50)	0.28
*L2:003	0.10 (82)	0.12 (111)	0.82 (0.61-1.11)	0.19
*L3:001	0.44 (366)	0.42 (393)	1.10 (0.91-1.32)	0.34
*L3:002	0.26 (216)	0.26 (248)	0.98 (0.79-1.21)	0.86
*L3:005	0.03 (29)	0.05 (44)	0.74 (0.46-1.19)	0.21
2DL5B				
*5B:002	0.11 (93)	0.11 (100)	1.06 (0.79-1.43)	0.70
Neg	0.88 (738)	0.89 (841)	0.94 (0.70-1.26)	0.68
**Centromeric *2DS3/5***				
*S3:001	0.11 (94)	0.11 (100)	1.07 (0.80-1.44)	0.65
Neg	0.88 (738)	0.89 (841)	0.94 (0.70-1.26)	0.68
2DP1				
*001	0.12 (97)	0.11 (105)	1.05 (0.79-1.41)	0.74
*002	0.43 (362)	0.40 (378)	1.15 (0.95-1.39)	0.15
*003	0.26 (216)	0.26 (243)	1.01 (0.82-1.25)	0.94
*016b	0.03 (27)	0.05 (45)	0.67 (0.42-1.09)	0.11
Neg	0.14 (116)	0.15 (141)	0.92 (0.71-1.20)	0.54
2DL1				

*001	0.03 (27)	0.05 (44)	0.69 (0.43-1.11)	0.13
*002	0.26 (219)	0.26 (249)	0.99(0.80-1.23)	0.95
*003	0.42 (351)	0.40 (380)	1.08 (0.89-1.30)	0.44
*004	0.11 (96)	0.10(93)	1.19 (0.88-1.61)	0.26
Neg	0.14 (119)	0.14 (131)	1.03 (0.79-1.35)	0.81
2DL4				
*001	0.28 (233)	0.26 (246)	1.10 (0.89-1.36)	0.37
*005	0.19 (158)	0.17 (162)	1.13 (0.89-1.44)	0.33
*006	0.03 (23)	0.04 (37)	0.70 (0.42-1.18)	0.18
*008	0.30 (248)	0.29 (274)	1.03 (0.84-1.27)	0.74
*011	0.20 (164)	0.18 (175)	1.08 (0.85-1.36)	0.55
3DL1				
*001	0.16 (132)	0.15 (145)	1.04 (0.80-1.34)	0.79
*002	0.12 (97)	0.12 (113)	0.97 (0.73-1.29)	0.83
*004	0.14 (117)	0.12 (116)	1.16 (0.88-1.53)	0.28
*005	0.18 (153)	0.17 (157)	1.13 (0.88-1.44)	0.34
*007	0.03 (23)	0.04 (38)	0.68 (0.41-1.14)	0.15
*008	0.06 (46)	0.05 (45)	1.17 (0.77-1.77)	0.47
*015	0.07 (58)	0.07 (70)	0.93 (0.65-1.34)	0.71
*020	0.03 (29)	0.02 (15)	2.20 (1.19-4.06)	0.01
Neg	0.20 (165)	0.21 (197)	0.94 (0.74-1.18)	0.57
3DS1				
*013	0.22 (185)	0.21 (199)	1.07 (0.85-1.34)	0.57
Neg	0.78 (651)	0.79 (747)	0.94 (0.75-1.17)	0.57
2DL5A				
*5A:001	0.16 (130)	0.16 (149)	0.99 (0.76-1.27)	0.91
*5A:005	0.05 (40)	0.04 (42)	1.08 (0.70-1.68)	0.72
Neg	0.80 (666)	0.80 (753)	1.00 (0.80-1.26)	0.97
**Telomeric** ***2DS3/5***				
*S3:001	0.01 (11)	0.01 (11)	1.13 (0.51-2.54)	0.76
*S3:002	0.03 (23)	0.03 (24)	1.09 (0.62-1.92)	0.77
*S5:002	0.15 (126)	0.16 (150)	0.94 (0.73-1.22)	0.65
Neg	0.80 (666)	0.80 (753)	1.00 (0.80-1.26)	0.97
2DS4				
*001	0.22 (185)	0.21 (200)	1.06 (0.85-1.33)	0.61
*003	0.22 (181)	0.22 (207)	0.99 (0.79-1.24)	0.91
*004	0.03 (24)	0.04 (39)	0.69 (0.42-1.15)	0.16
*006	0.14 (115)	0.13 (125)	1.05 (0.80-1.37)	0.74
*010	0.19 (161)	0.18 (174)	1.06 (0.83-1.34)	0.64
Neg	0.20 (168)	0.21 (201)	0.93 (0.74-1.17)	0.55
2DS1				

*002	0.18 (149)	0.19 (180)	0.92 (0.73-1.17)	0.51
Neg	0.80 (669)	0.80 (759)	0.99 (0.78-1.25)	0.91
3DL2				
*001	0.29 (239)	0.25 (237)	1.20 (0.97-1.48)	0.09
*002	0.18 (154)	0.20 (186)	0.92 (0.73-1.17)	0.51
*003	0.06 (51)	0.07 (65)	0.88 (0.61-1.29	0.51
*005	0.05 (45)	0.06 (57)	0.89 (0.60-1.32)	0.56
*007	0.19 (160)	0.18 (173)	1.06 (0.83-1.34)	0.64
*008	0.03 (24)	0.04 (38)	0.71 (0.43-1.19)	0.19
*009	0.09 (75)	0.06 (56)	1.56 (1.09-2.23)	0.01
*010	0.04 (30)	0.08 (71)	0.46 (0.30-0.71)	0.0005
*011	0.03 (27)	0.02 (18)	1.71 (0.95-3.08)	0.08

Duplicated alleles were included as distinct loci, frequencies for these are not shown

Supplementary table 3 : Carrier frequencies of HLA ligands

	Cases % (N=404)	Controls % (N=473)	OR (95%CI)	*P*
**Bw4 (HLA-A and -B based)**				
**0**	28.7 (116)	33.4 (158)	0.80 (0.60-1.07)	0.14
**1**	46.5 (188)	41.4 (196)	1.23 (0.94-1.61)	0.13
**2**	21.3 (86)	20.7 (98)	1.04 (0.75-1.43)	0.83
**3**	3.2 (13)	4.2 (20)	0.76 (0.38-1.52)	0.44
**4**	0.2 (1)	0.2 (1)	1.17 (0.16-8.35)	0.87
**Bw4 (HLA-B based)**				
**0**	41.6 (168)	44.6 (211)	0.88 (0.68-1.16)	0.37
**1**	49.5 (200)	45.5 (215)	1.18 (0.90-1.53)	0.23
**2**	8.9 (36)	9.9 (47)	0.89 (0.57-1.40)	0.61
**C1**				
**0**	11.1 (45)	11.8 (56)	0.94 (0.62-1.41)	0.75
**1**	42.8 (173)	44.2 (209)	0.95 (0.72-1.24)	0.69
**2**	46.0 (186)	44.0 (208)	1.09 (0.83-1.42)	0.54
**C2**				
**0**	46.3 (187)	44.0 (208)	1.10 (0.84-1.43)	0.49
**1**	42.6 (172)	44.2 (209)	0.94 (0.72-1.22)	0.63
**2**	11.1 (45)	11.8 (56)	0.94 (0.62-1.41)	0.75
**A3/11**				
**0**	62.4 (252)	60.9 (288)	1.06 (0.81-1.40)	0.65
**1**	32.7 (132)	34.2 (162)	0.93 (0.70-1.23)	0.62
**2**	5.0 (20)	4.9 (23)	1.02 (0.56-1.86)	0.94

Numbers 0-4 represent the number of ligands present in each individual

Supplementary table 4: Carrier frequencies of KIR-HLA pairs

	Cases % (N=404)	Controls % (N=473)	OR (95%CI)	*P*
**KIR3DL1 and Bw4 (HLA-A and -B based)**				
	67.8 (274)	64.3 (304)	1.17 (0.88-1.55)	0.27
**KIR3DL1 and Bw4 (HLA-B based)**				
	55.7 (225)	53.5 (253)	1.09 (0.84-1.43)	0.51
**KIR3DL1 and Bw4^{80I} (HLA-B based)**				
	20.0 (81)	13.5 (64)	1.60 (1.12-1.28)	0.01
**KIR3DL1 and Bw4^{80T} (HLA-B based)**				
	39.6 (160)	43.6 (206)	0.85 (0.65-1.11)	0.24
**KIR2DL2 and C1**				
	39.4 (159)	39.7 (188)	0.98 (0.75-1.29)	0.91
**KIR2DL3 and C1**				
	83.4 (337)	82.7 (391)	1.05 (0.74-1.50)	0.77
**KIR2DS2 and C1**				
	39.9 (161)	40.4 (191)	0.98 (0.75-1.28)	0.87
**KIR2DL1 and C2**				
	52.5 (212)	54.8 (259)	0.91 (0.70-1.19)	0.50
**KIR2DL2 and C2**				
	24.8 (100)	25.2 (119)	0.98 (0.71-1.33)	0.89
**KIR2DS1 and C2**				
	25.2 (102)	25.4 (120)	0.99 (0.73-1.35)	0.97
**KIR3DL2 and A3/11**				
	37.6 (152)	39.1 (185)	0.94 (0.72-1.23)	0.65

Supplementary table 5: Carrier frequencies for haplotype motif combinations

Haplotype combination	Cases % (N=418)	Controls % (N=473)	OR (95%CI)	*P*
**Centromeric motif**				
**A/A**	55.0 (230)	53.5 (253)	1.1 (0.82-1.38)	0.65
**A/B**	36.6 (153)	33.8 (160)	1.1 (0.86-1.49)	0.39
**B/B**	6.0 (25)	6.3 (30)	0.9 (0.55-1.61)	0.83
**Any/DelDup***	2.4 (10)	6.3 (30)	0.4 (0.22-0.75)	0.006
**Telomeric motif**				
**A/A**	60.0 (251)	59.4 (281)	1.0 (0.79-1.34)	0.85
**A/B**	28.0 (115)	27.7 (131)	1.0 (0.74-1.33)	0.95
**B/B**	4.0 (15)	2.7 (13)	1.3 (0.63-2.72)	0.47
**Any/DelDup***	8.9 (37)	10.1 (48)	0.9 (0.55-1.35)	0.51

**The haplotype combination consists of several different haplotypes and haplotype combinations; KIR-A or KIR-B in combination with at least one haplotype containing duplicated or deleted genes. Notably, the assigning of haplotypes containing duplication or deletion of genes may be ambiguous.*

Haplotypes for the centromeric and the telomeric regions were assigned as haplotype A or B based on the gene content for each individual prior to frequency calculations of the haplotype combinations. For the centromeric region haplotype B represents the two sub-haplotypes cB01 and cB02.
